# In Contagious Diseases

**Published:** 1891-12

**Authors:** C. B. Gibson


### IN CONTAGIOUS DISEASES.

In case of contagious diseases, the sick room may be more surely and completely disinfected by the use of chlorine. The gas may be prepared in this manner: Procure at a drug store a few ounces of manganese dioxide—black oxide of manganese—and about twice the quantity of concentrated hydrochloric (muriatic) acid. Pour on to a large plate or platter the black oxide. Place the platter on blocks of wood or bricks built up high enough to admit of a small kerosene lamp being placed under it, the top considerably below the bottom of the platter.

Arrange another lamp with a basin of water over it to furnish moisture, or fill the room with steam beforehand. When every thing has been arranged, light the lamps, turn the one under the platter low enough so as not to heat too rapidly or too hot, pour on enough of the hydrochloric acid to make a thin mixture, stir well with the black oxide, using a stick, close the room tightly and let remain so for six or eight hours. Of course the room must be closed and calked up completely the same as in the use of brimstone. Care should be taken not to overheat the platter to avoid breaking. The furnishings of rooms may be injured by the chlorine, for it attacks fabrics, coloring matter and the metals, but is a sure, safe and complete disinfectant and germ destroyer.

Chlorine was first employed in this manner by Professor R. Ogden Doremus in the disinfecting of the lying-in hospital of the Bellevue Hospital Medical College, New York, and was the most successful attempt ever made. The building had become valueless, all the patients suffered from puerperal fever. After being disinfected not a case occurred.

Cess-pools, vaults and utensils used in sick rooms may be both deodorized and disinfected by the use of bleaching powder or similar preparations, in sufficient quantities, but the air cannot be disinfected completely by placing any of them around in dishes. Chlorine or bromine will not destroy germs unless present in quantities which render the air unfit for breathing.—C. B. Gibson, M. D.

